# Capping proteins regulate fungal development, DON‐toxisome formation and virulence in *Fusarium graminearum*

**DOI:** 10.1111/mpp.12887

**Published:** 2019-11-06

**Authors:** Guangfei Tang, Ahai Chen, Dawood H. Dawood, Jingting Liang, Yun Chen, Zhonghua Ma

**Affiliations:** ^1^ State Key Laboratory of Rice Biology Institute of Biotechnology Zhejiang University Hangzhou 310058 China; ^2^ Key Laboratory of Molecular Biology of Crop Pathogens and Insects Zhejiang University Hangzhou 310058 China; ^3^ Department of Agriculture Chemistry Faculty of Agriculture Mansoura University Mansoura 35516 Egypt

**Keywords:** actin cytoskeleton, capping protein, deoxynivalenol (DON), *Fusarium graminearum*, toxisome, virulence

## Abstract

Deoxynivalenol (DON) is an important trichothecene mycotoxin produced by the cereal pathogen *Fusarium graminearum*. DON is synthesized in organized endoplasmic reticulum structures called toxisomes. However, the mechanism for toxisome formation and the components of toxisomes are not yet fully understood. In a previous study, we found that myosin I (FgMyo1)‐actin cytoskeleton participated in toxisome formation. In the current study, we identified two new components of toxisomes, the actin capping proteins (CAPs) FgCapA and FgCapB. These two CAPs form a heterodimer in *F. graminearum*, and physically interact with FgMyo1 and Tri1. The deletion mutants Δ*FgcapA* and Δ*FgcapB* and the double deletion mutant ΔΔ*FgcapA/B* dramatically reduced hyphal growth, asexual and sexual reproduction and endocytosis. More importantly, the deletion mutants markedly disrupted toxisome formation and DON production, and attenuated virulence *in planta*. Collectively, these results suggest that the actin CAPs are associated with toxisome formation and contribute to the virulence and development of *F. graminearum*.

## Introduction

The actin cytoskeleton is crucial for various eukaryotic cellular processes, such as cell division, cytokinesis, endocytosis, vesicle trafficking and motility (Pollard and Borisy, 2003; Pollard and Cooper, 2009). For the progression of these cellular processes, cells must rapidly regulate the turnover between the monomeric form of actin and its filamentous form, F‐actin, in response to environmental stimuli (Cooper and Schafer, 2000; Rohatgi *et al.*, 1999). A number of actin‐interacting or related proteins are involved in modulating actin dynamics, including the actin‐depolymerizing factor (ADF)/cofilin family, Arp2/3 complex and capping proteins (CAPs) (Cooper and Schafer, 2000).

Actin filaments are polar double‐helical polymers of globular subunits and have two ends, referred to as the barbed and pointed ends. The barbed end, compared with the pointed end, plays a major role in filamentous dynamics, as it has higher association and dissociation rate constants for actin subunits (Wear and Cooper, 2004). Specialized proteins bind to the ends of actin to regulate the assembly and disassembly of actin filaments (Cooper and Schafer, 2000; dos Remedios *et al.*, 2003). CAPs are important for the assembly of various actin structures by tightly capping the barbed end of actin filaments to prevent the addition or loss of actin subunits (Cooper and Schafer, 2000; Shekhar *et al.*, 2016; Wear and Cooper, 2004). CAPs are ubiquitous and highly conserved in all eukaryotic organisms from yeasts to mammals. CAPs exist in cells in a stable heterodimeric form consisting of alpha (α) and beta (β) subunits that have similar secondary structures but lack sequence similarity (Cooper and Sept, 2008; Wear and Cooper, 2004). Studies have demonstrated that deletion mutants or loss‐of‐function of CAPs cause defects in several cellular and developmental processes in various organisms. CAPs participate in stereocilia widening by preventing newly elongated actin filaments from depolymerizing in mice (Avenarius *et al.*, 2017). Silencing CAPs in both cultured mammalian B16F10 cells and neurons of developing neocortices impairs cell migration (Sinnar *et al.*, 2014). In the apicomplexan parasite *Plasmodium*, the β subunit of the CAP is an essential regulator of sporozoite motility and malaria transmission (Ganter *et al.*, 2009). In *Arabidopsis,* CAP mutants have an abnormal cell morphology and have 10–20% longer hypocotyls than wild type (Li *et al.*, 2012). In *Saccharomyces cerevisiae*, deletion of either the *CAP1* or *CAP2* gene leads to an abnormal actin distribution with fewer actin cables and an increased number of actin patches. The mutant cells appear round and enlarged and exhibit growth defects with a heterogeneous size distribution (Amatruda *et al.*, 1990; Kovar *et al.*, 2005). In the plant pathogenic fungus *Magnaporthe oryzae*, the CAP homologues MoCapA and MoCapB are important for endocytosis and actin dynamics and are directly linked to fungal growth, conidiation and pathogenicity (Li *et al.*, 2017b).

Fusarium head blight (FHB), predominately caused by *Fusarium graminearum*, is one of the most devastating diseases of wheat worldwide (Xu and Nicholson, 2009). FHB epidemics cause yield losses in FHB‐prone regions of the world (Nganje *et al.*, 2004). In addition to severe yield losses, FHB leads to harmful mycotoxin contamination in infested grains, such as deoxynivalenol (DON), nivalenol and zearalenone (Chen *et al.*, 2019). Among them, DON is the most frequently detected mycotoxin in cereal grains (Lee and Ryu, 2017). The biosynthetic enzymes and regulators encoded by 15 *TRI* genes have been well characterized (Alexander *et al.*, 2009; Gale *et al.*, 2005). Under DON induction conditions *in vitro* and during the infection process *in planta*, the mRNA expression of *TRI* genes is highly induced, and subsequently translated proteins localize on a perinuclear organized smooth endoplasmic reticulum (OSER). The OSER is considered the DON biosynthesis compartment and designated the ‘DON‐toxisome’ (hereafter called ‘toxisome’) (Boenisch *et al.*, 2017). Recently, we found that the myosin I‐actin cytoskeleton participates in DON‐toxisome formation and the production of DON in *F. graminearum* (Tang *et al.*, 2018).

Although the myosin‐actin cytoskeleton has been suggested to play an important role in DON biosynthesis, the actin *CAP* genes have not yet been functionally characterized in *F. graminearum*. Additionally, our previous study found that CAPs were captured using either FgMyo1 or the DON biosynthetic enzyme Tri1 as a bait in affinity capture‐mass spectrometry assays under DON‐inducing conditions (Tang *et al.*, 2018). This prompted us to decipher the biological function of CAPs in DON biosynthesis. In this study, two *CAP* genes were identified and genetically analysed in *F. graminearum*. The results indicate that CAPs play an important role in mycelial growth, toxisome formation, DON biosynthesis and virulence in *F. graminearum.*

## Results

### Capping proteins interact with Tri1 and FgMyo1 in *F. graminearum*

The toxisome is considered the DON biosynthesis compartment in *F. graminearum*, while proteins that participate in toxisome formation are still under investigation (Boenisch *et al.*, 2017). DON biosynthetic enzymes such as Tri1, Tri4 and the class I myosin FgMyo1 have been identified as components of the toxisome (Tang *et al.*, 2018). To identify other proteins involved in toxisome formation, we previously performed affinity capture‐mass spectrometry (AC‐MS) assays using Tri1 and FgMyo1 as baits (Tang *et al.*, 2018). Two proteins (loci FGSG_08621 and FGSG_01226) encoding homologues to fungal F‐actin CAP subunits were captured in both AC‐MS assays. A phylogenetic analysis of the putative CAPs, including *F. graminearum* and seven other tested fungi, demonstrated that the fungal CAP α and β subunits were highly conserved. Interestingly, the CAP subunit homologues in filamentous fungi and yeasts were notably divided into two groups. Based on the phylogenetic tree, we named FGSG_08621 FgCapA (α subunit of CAP) and FGSG_01226 FgCapB (β subunit of CAP) in *F. graminearum* (Fig. S1).

**Figure 1:**
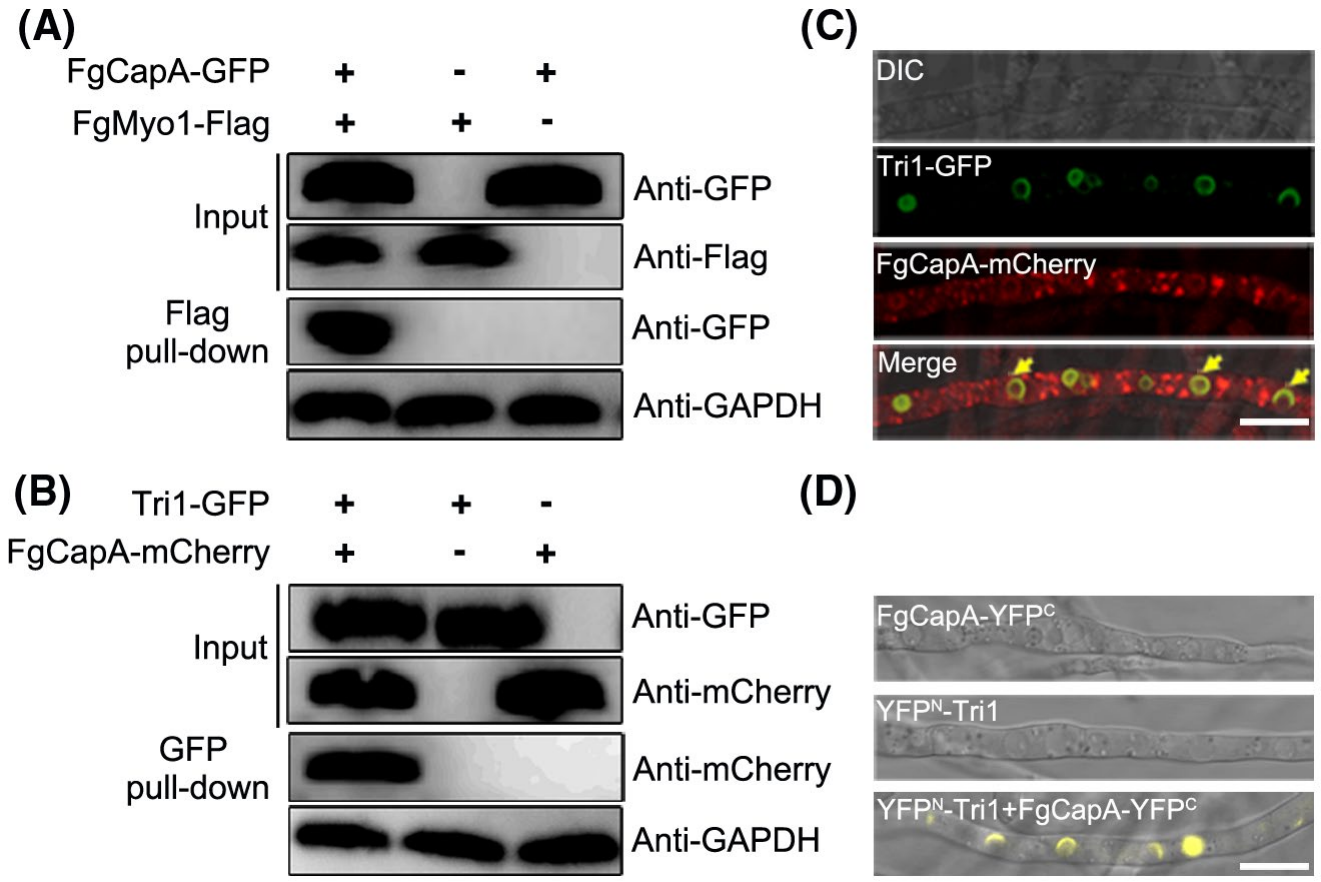
FgCapA interacts with FgMyoI and Tri1. (A) The interaction of FgCapA‐GFP and FgMyo1‐Flag was verified by the co‐immunoprecipitation (Co‐IP) assay. Total protein (Input) extracted from the strain bearing FgCapA‐GFP and FgMyo1‐Flag constructs or a single construct (FgCapA‐GFP or FgMyo1‐Flag) were subjected to SDS‐PAGE, and immunoblots were incubated with anti‐FLAG and anti‐GFP antibodies, as indicated (Input panel). Each protein sample was pulled down using anti‐Flag agarose and further detected with anti‐GFP antibody (Flag pull‐down panel). Protein samples were also detected with anti‐GAPDH antibody as a reference. (B) The interaction of FgCapA‐mCherry and Tri1‐GFP was verified by the Co‐IP assay. Protein samples were pulled down using anti‐GFP agarose and further detected with an anti‐mCherry antibody. Protein samples were also detected with anti‐GAPDH antibody as a reference. (C) FgCapA‐mCherry was partially colocalized with Tri1‐GFP on DON‐toxisomes at 48 h of incubation in trichothecene biosynthesis induction (TBI) medium. Localization is indicated with yellow arrows. Bar = 10 µm. (D) The interaction of FgCapA with Tri1 was confirmed by bimolecular fluorescence complementation (BiFC) assay. The constructs of YFP^N^‐Tri1 and pFgCapA‐YFP^C^ were co‐transformed into *Fusarium graminearum* PH‐1 to generate the strain YFPN ‐Tri1 + FgCapA‐YFP^C^. The strains bearing a single construct (YFP^N^‐Tri1 or FgCapA‐YFP^C^) were used as negative controls. The yellow fluorescent protein (YFP) signals in hyphae of each strain grown in the TBI medium were examined under a confocal microscope. DIC, differential interference contrast. Bar = 10 μm.

To further determine the interaction patterns between FgCAPs and FgMyo1 or FgTri1, we conducted coimmunoprecipitation (Co‐IP), colocalization and bimolecular fluorescence complementation (BiFC) assays under DON‐inducing conditions. As shown in Fig. 1A,B, FgCapA interacted with FgMyo1 and Tri1 in the Co‐IP assay. A strain bearing FgCapA‐mCherry (red fluorescent protein) and Tri1‐GFP (green fluorescent protein) was constructed in the wild‐type background and cultured in liquid trichothecene biosynthesis induction (TBI) medium to observe colocalization. The red fluorescence signals (FgCapA‐mCherry) were diffuse throughout the cytoplasm and partially colocalized with Tri1‐GFP at the toxisome (Fig. 1C). Moreover, the direct interaction between FgCapA and Tri1 was verified by BiFC (Fig. 1D). Similar to FgCapA, FgCapB also interacted with FgMyo1 and Tri1 (Fig. S2). Combining the AC‐MS, Co‐IP, colocalization and BiFC assays, these results indicate that CAPs interact with Tri1 and FgMyo1 in *F. graminearum.*

### FgCapA and FgCapB form a heterodimer

FgCapA and FgCapB are predicted to encode proteins of 316 and 282 amino acids and share 37% and 45% sequence identity, respectively, with counterparts in *S. cerevisiae*. However, FgCapA and FgCapB share only 10% amino acid sequence identity.

**Figure 2:**
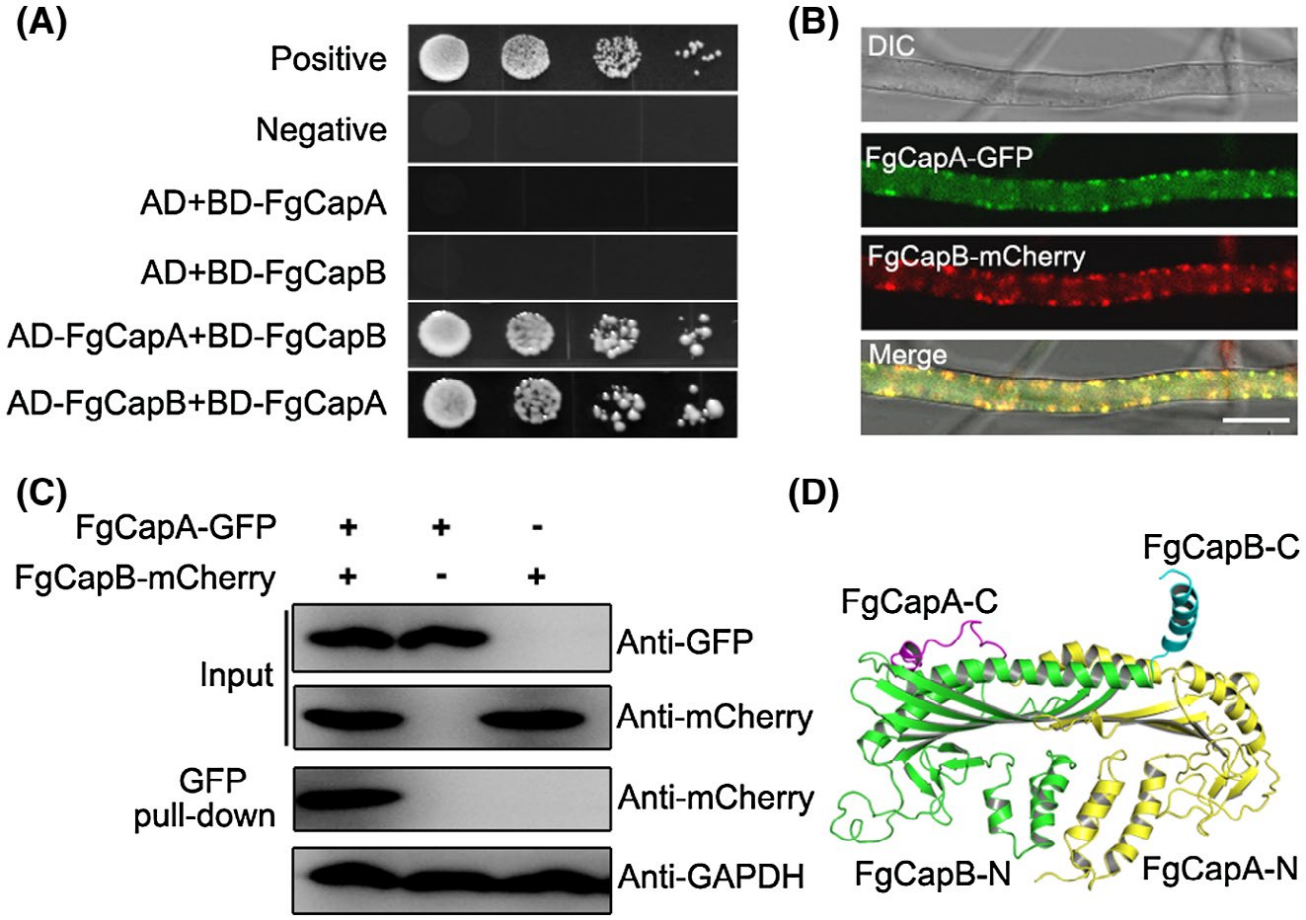
FgCapA and FgCapB form a heterodimer. (A) The CAPs interacted with each other in the yeast‐two‐hybrid assay. Serial concentrations of yeast cells transferred with the bait and prey constructs indicated in the figure were assayed for growth on SD−Leu−Trp−His−Ade plates. pGBKT7‐53 and pGADT7 were used as positive controls. Another pair of plasmids, pGBKT7‐Lam and pGADT7, were used as negative controls. Images were taken after 3 days of incubation at 30 °C. pGADT7 and pGBKT7 are abbreviated to AD and BD, respectively. (B) FgCapA‐GFP colocalized with FgCapB‐mCherry. Vegetative hyphae of dual‐labelled strains were observed under a confocal microscope after incubation in PDB medium for 24 h. DIC, differential interference contrast. Bar = 10 µm. (C) The interaction of FgCapA‐GFP and FgcapB‐mCherry was verified by the Co‐IP assay. Protein samples were pulled down using anti‐GFP agarose and further detected with an anti‐mCherry antibody. Protein samples were also detected with anti‐GAPDH antibody as a reference. (D) A three‐dimensional homology model of the *Fusarium graminearum* CAP heterodimer based on the structure of chicken CAPs (Protein Data Bank, accession code 1IZN).

It has been shown that CAPs exist in a more stable heterodimer form consisting of α and β subunits in cells. To test whether FgCapA and FgCapB also interact with each other to form a heterodimer in *F. graminearum*, a yeast two‐hybrid assay was performed. The results indicated that FgCapA and FgCapB indeed interact with each other (Fig. 2A). Subsequently, the localization patterns of FgCapA and FgCapB were visualized by confocal microscopy. The *FgCAPA‐GFP* and *FgCAPB‐mCherry* fusion constructs were co‐transformed into the wild‐type strain. As shown in Fig. 2B, the FgCapA‐GFP and FgCapB‐mCherry fused proteins were mainly distributed in a similar patch pattern in hyphae grown in potato dextrose broth (PDB) medium. Importantly, the colocalization of FgCapA‐GFP and FgCapB‐mCherry was clearly observed. Additionally, a Co‐IP assay showed that FgCapA and FgCapB interact with each other (Fig. 2C). A homology model of the *F. graminearum* CAP, based on the structure of the chicken CAP (Yamashita *et al.*, 2003), yielded a mushroom‐like structure (Fig. 2D). The stalk was formed by a six α‐helix bundle (three α‐helices from N‐terminal regions of each subunit). The mushroom cap consisted of a ten‐stranded anti‐parallel β‐sheet (five strands from each subunit), on top of which was two long C‐terminal α‐helices (one helix from each subunit) running perpendicular to the β‐sheet strands (Fig. 2D).

### CAPs interact with actin and participate in actin organization

**Figure 3:**
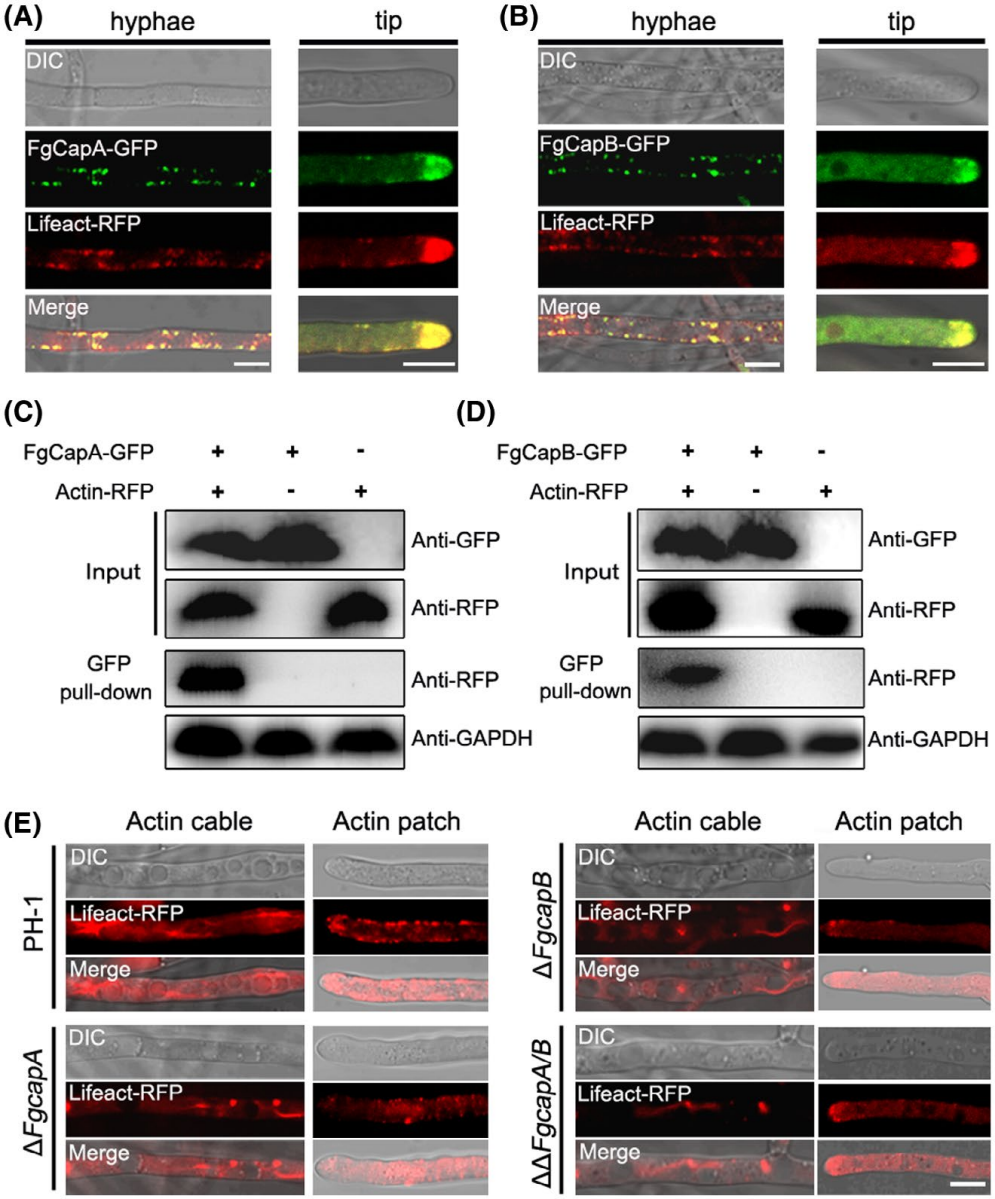
Capping proteins interact with actin for actin organization in *Fusarium graminearum*. (A) and (B) FgCapA‐GFP and FgCapB‐GFP colocalized with the F‐actin reporter Lifeact‐RFP in the mature hyphae and hyphal tips. Vegetative hyphae of dual‐labelled strains were observed under a confocal microscope after incubation in potato dextrose broth (PDB) medium for 24 h. DIC, differential interference contrast. Bar = 10 µm. (C) and (D) The interaction of FgCapA‐GFP or FgCapB‐GFP and actin‐RFP was verified by the Co‐IP assay. Protein samples were pulled down using anti‐GFP agarose and further detected with an anti‐RFP antibody. Protein samples were also detected with anti‐GAPDH antibody as a reference. (E) Deletion of FgCapA or FgCapB affected the actin morphology. Actin cable and patch was observed by expressing Lifeact‐RFP in the wild‐type and mutants. Hyphae of labelled strains were grown in PDB for 24 h. Representative micrographs of actin patterns in each strain are shown. Bar = 10 µm.

CAPs bind the ends of actin filaments and play a critical role in regulating the addition and dissociation of actin subunits (Rao *et al.*, 2014). Therefore, we determined whether the FgCAPs also interacted with actin. First, the colocalization patterns were observed in the wild‐type strain dual‐labelled with FgCapA‐GFP or FgCapB‐GFP and the actin reporter, Lifeact‐RFP. As shown in Fig. 3A,B, signals of FgCap‐GFP mostly colocalized with the red fluorescence signals of actin patches, especially in the cells of hyphal tips, suggesting that the FgCaps potentially interacted with actin. Next, we conducted Co‐IP assays and confirmed that the CAPs interacted with actin in *F. graminearum* (Fig. 3C,D).

To investigate whether or not CAPs are involved in the actin organization, we constructed deletion mutants Δ*FgcapA*, Δ*FgcapB* and double‐mutant Δ*FgcapA/B* in the wild‐type strain expressing the actin reporter, Lifeact‐RFP, and observed the actin patterns in these strains. Most of the wild‐type strain generally showed several long actin cables in the hyphae, while fewer and shorter cables were formed in the hyphae of mutants (Fig. 3E). Meanwhile, the actin patches were reduced at the top of the mutant hyphae compared with wild type (Fig. 3E). Taken together, FgCapA and FgCapB physically form a heterodimer, interact with actin and participate in actin organization in *F. graminearum*.

### FgCaps are important for hyphal growth and fungal reproduction

**Figure 4:**
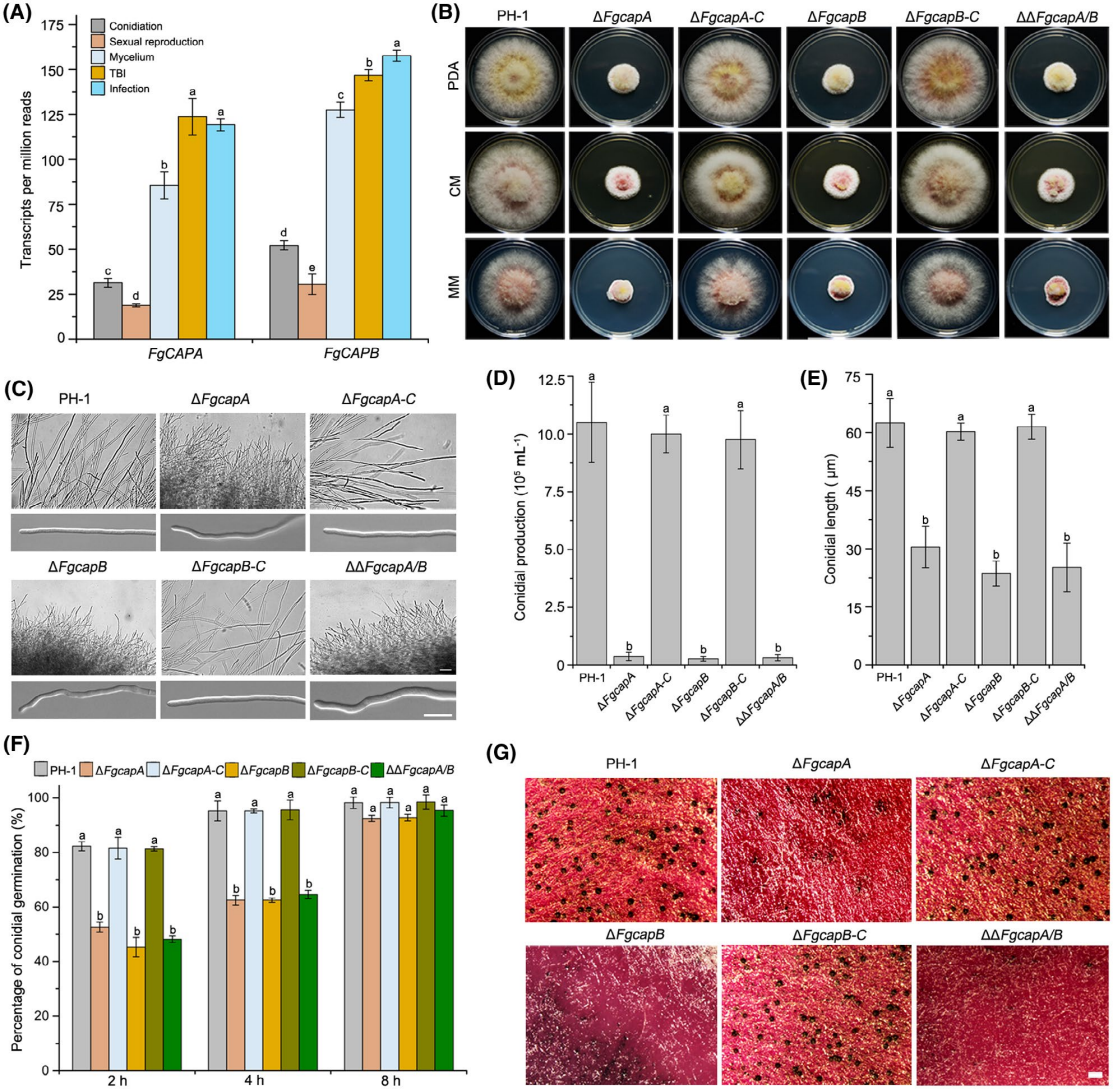
Capping proteins are required for vegetative growth, asexual and sexual reproduction in *Fusarium graminearum*. (A) Transcriptional levels of the *FgCAPA* and *FgCAPB* genes in conidiation (carboxymethyl cellulose, CMC), sexual reproduction (carrot agar, CA), mycelium (potato dextrose agar, PDA), and trichothecene biosynthesis induction (TBI) media and during the plant infection process by RNA‐Seq. (B) Colony morphology of the wild‐type *F. graminearum* PH‐1, Δ*FgcapA*, Δ*FgcapB* and ΔΔ*FgcapA/B* grown on PDA, CMC and minimal medium (MM) agar plates for 3 days at 25 °C. (C) Hyphal branching patterns and tip growth of the wild‐type and mutant strains grown on PDA for 1 day. Bar = 100 µm. (D) Conidial production of the wild‐type, mutant and complementation strains harvested from 4‐day‐old CMC cultures. (E) Average length of conidia of the wild‐type, mutant and complementation strains. Conidia produced by each strain were harvested after incubation in CMC medium for 4 days. Length of conidia was measured using the Nikon NIS‐Element D 4. 20 imaging software (*n* = 50 for each strain). (F) Conidial germination of the wild‐type, mutant and complementation strains in 2% sucrose solution. Bars denote standard deviations from three experiments. Columns labelled with the same letter are not significantly different according to the least significant difference (LSD) test at *P* = 0.05. (G) CAP mutants reduced the fungal sexual development. Strains grown on CA were self‐fertilized. Photographs of perithecia were taken after 3 weeks of incubation. Bar = 200 µm.

Our in‐house RNA‐sequencing (RNA‐Seq) data indicated that *FgCAPA* and *FgCAPB* had similar transcriptional patterns in all five tested conditions, including on conidiation medium (carboxymethyl cellulose, CMC), carrot agar (sexual reproduction), PDB, TBI and during plant infection. Notably, their expression was increased under DON‐inducing conditions and *in planta* (Fig. 4A).

To characterize the function of FgCapA and FgCapB, we constructed single and double deletion mutants, Δ*FgcapA*, Δ*FgcapB* and ΔΔ*FgcapA/B* in the wild‐type strain using homologous recombination. The deletion mutants were identified by polymerase chain reaction (PCR) amplification and Southern blot analysis (Fig. S3). At least three independent transformants of the Δ*FgcapA*, Δ*FgcapB* and ΔΔ*FgcapA/B* mutants were obtained, and all transformants showed similar defective phenotypes under the tested conditions. Therefore, the phenotypes of one representative transformant for each mutant are shown in the following experiments. To confirm that phenotypic defects of mutants were directly related to the deletion, we complemented single deletion mutants with the corresponding open reading frame (ORF) fused with *gfp*, encoding green fluorescent protein (GFP), at the carboxyl terminus under the native promoter and generated the complementation strains Δ*FgcapA*‐C (*FgcapA::*P*_CAPA_FgCAPA‐GFP*) and Δ*FgcapB*‐C (Δ*FgcapB::*P*_CAPB_FgCAPB‐GFP*).

The CAP mutants demonstrated obvious hyphal growth defects on CM, PDA and minimal medium (MM) compared with the wild‐type strain PH‐1. The mutants grew significantly slower and with more compacted aerial hyphae than the wild‐type strain (Figs 4B and S4A). Furthermore, microscopic observation showed that the hyphae of the mutants were highly branched and misshapen (Fig. 4C).

The CAP mutants produced fewer conidia than the wild‐type strain after 4 days of incubation in CMC. Quantification data showed that the Δ*FgcapA* and Δ*FgcapB* mutants produced 10^4^ conidia per millilitre, which was 30 times fewer than that produced by the wild‐type strain (Fig. 4D). To further examine conidial morphology, conidia were stained with calcofluor white (CFW) and observed under a fluorescence microscope. The average size of the conidia produced by the mutants was shorter than that of the wild‐type strain (Fig. 4E). Moreover, the conidia of the mutants harboured fewer septa. Most of the mutant conidia (75%) had only two or three septa, whereas the majority of conidia produced by the wild‐type strain had five septa (Fig. S4B,C). Meanwhile, the abnormal conidia of the mutants germinated more slowly than the conidia of the wild‐type strain in the presence of 2% sucrose (Fig. 4F). Moreover, the Δ*FgcapA* and Δ*FgcapB* mutants formed fewer perithecia compared with the wild‐type (Fig. 4G). The double ΔΔ*FgcapA/B* mutant showed similar phenotypes to the single gene mutant. Phenotypic defects of mutants were completely rescued in complementation strains. Thus, our results indicate that both FgCapA and FgCapB are critical for hyphal growth, asexual and sexual reproduction in this fungus.

### FgCaps are required for endocytosis and adaption to abiotic stress

**Figure 5:**
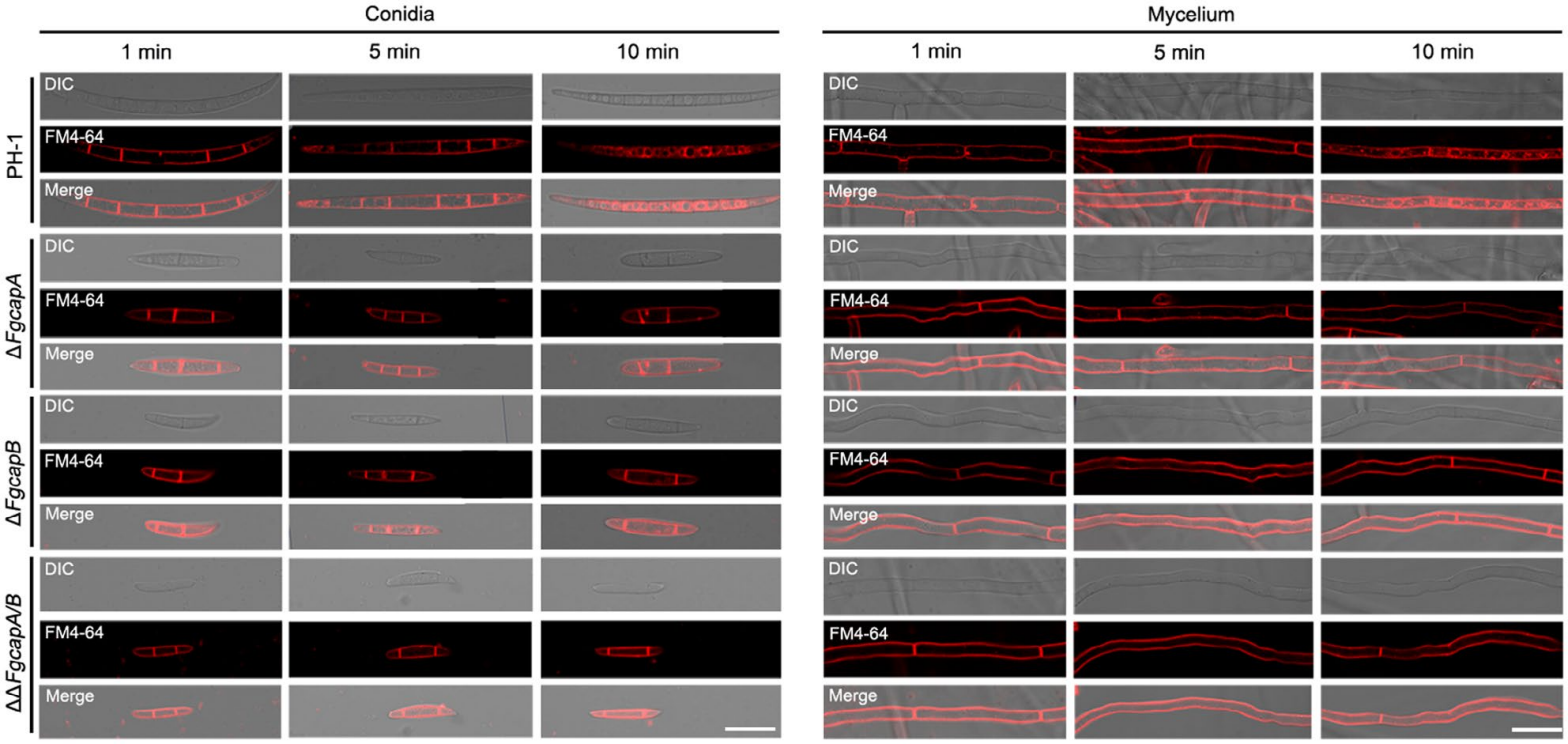
*FgCAP* gene deletion mutants have reduced endocytosis. Time‐course of FM4‐64 internalization via the endocytic pathway in mycelium and conidia of *Fusarium graminearum* wild‐type PH‐1 and mutant strains. Bar = 10 µm.

In a previous study, we found that deletion mutants of the actin cytoskeleton‐related genes *FgPRK1* and *FgEND3* caused endocytic defects in *F. graminearum* (Tang *et al.*, 2018). Given that the FgCaps interacted with actin, we tested whether deletion of the *CAP* genes also affected the endocytosis process using the FM4‐64 staining assay. The plasma membrane and septa were quickly stained with FM4‐64 in both the conidia and mycelia of all tested strains. The FM4‐64 dye was endocytosed and generated clear fluorescence signals on the membrane of intracellular organelles, such as vacuoles and endosomes, after a 10 min staining of the wild‐type conidia and mycelia. However, the endocytosis of the fluorescent dye was dramatically hindered in all mutants under the same conditions (Fig. 5). These results suggest that the FgCap proteins are important for the endocytosis process in *F. graminearum*.

**Figure 6:**
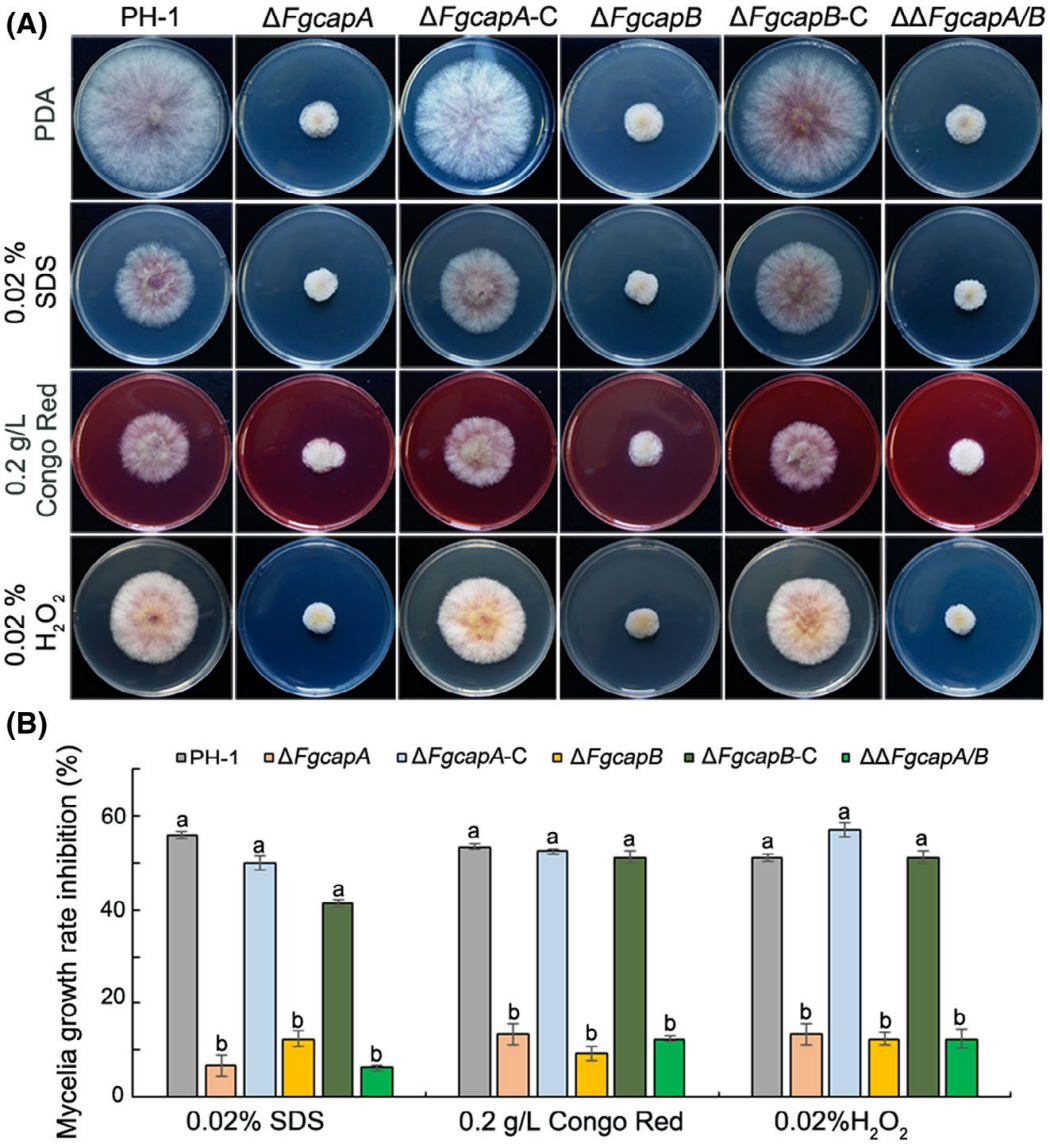
*FgCAP* gene deletion mutants have reduced sensitivities to abiotic stresses. (A) The sensitivity of *Fusarium graminearum* wild‐type PH‐1, mutant and complementation strains to 0.02% SDS (cell membrane‐damaging agent) and Congo red (cell wall‐damaging agent) at the final concentration of 0.2 g/L and 0.02% H_2_O_2_. (B) The inhibition of the mycelial growth rate was examined after each strain was incubated for 3 days on potato dextrose agar (PDA) supplemented with each stress compound. Bars denote standard deviations from three experiments. Columns labelled with the same letter are not significantly different according to the LSD test at *P* = 0.05.

Eukaryotic cells rapidly regulate the turnover of actin forms in response to environmental stimuli, which require actin CAPs (Cooper and Schafer, 2000; Rohatgi *et al.*, 1999). Therefore, we were interested in determining the sensitivities of the *FgCAP* gene mutants to various abiotic stresses, including cell membrane stress, cell wall and oxidative stresses generated by sodium dodecyl sulphate (SDS), Congo red and H_2_O_2_, respectively. The sensitivity assays showed that the CAP mutants demonstrated significantly decreased sensitivity to all three tested abiotic agents compared to the wild‐type strain PH‐1 (Fig. 6). The complementation strains Δ*FgcapA*‐C and Δ*FgcapB*‐C exhibited similar sensitivities to that of the wild‐type strain (Fig. 6), indicating that the complementation strains successfully rescued the defects of the corresponding mutant to abiotic stresses.

### F‐actin‐capping motifs are essential for the function of FgCapA but not FgCapB

**Figure 7:**
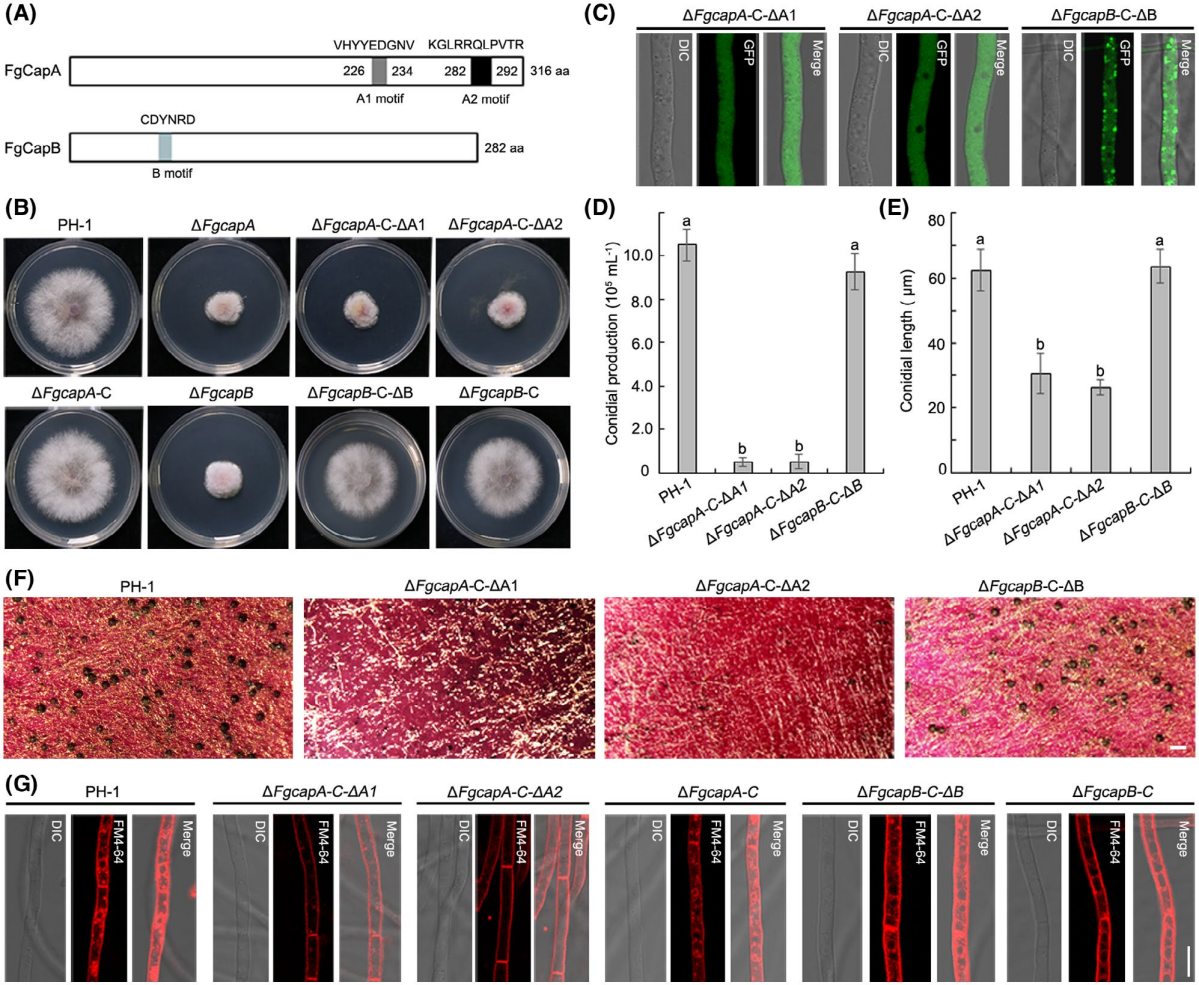
F‐actin‐capping motifs are essential for the function of FgCapA. (A) Schematic architecture of FgCapA and FgCapB. The F‐actin‐capping motifs and their corresponding amino acid sequences are indicated. (B) F‐actin‐capping motifs were indispensable for FgCapA function on vegetative growth, but not for FgCapB. *Fusarium graminearum* wild‐type PH‐1, Δ*FgcapA* and the complementation strains Δ*FgcapA*‐C, Δ*FgcapA*‐C‐ΔA1 (lacking the F‐actin‐capping motif A1) and Δ*FgcapA*‐C‐ΔA2 (lacking the F‐actin‐capping motif A2); Δ*FgcapB,* Δ*FgcapB‐C* and Δ*FgcapB*‐C‐ΔB (lacking the F‐actin‐capping motif B) were grown on potato dextrose agar (PDA) for 3 days before imaging. (C) The subcellular localization of F‐actin‐capping motif truncated mutants, Δ*FgcapA*‐C‐ΔA1, Δ*FgcapA*‐C‐ΔA2 and Δ*FgcapB*‐C‐ΔB in the mycelia grown in potato dextrose broth (PDB). Bar = 10 µm. (D) Conidial production of the wild‐type, F‐actin‐capping motif truncated mutants, Δ*FgcapA*‐C‐ΔA1, Δ*FgcapA*‐C‐ΔA2 and Δ*FgcapB*‐C‐ΔB harvested from 4‐day‐old carboxymethyl cellulose (CMC) cultures. (E) Average length of conidia of the wild‐type and F‐actin‐capping motif truncated mutants. Conidia produced by each strain were harvested after incubation in CMC medium for 4 days. Length of conidia was measured using the Nikon NIS‐Element D 4. 20 imaging software (*n* = 50 for each strain). (F) Perithecium formation of F‐actin‐capping motif truncated mutants on carrot agar medium. Photographs of perithecia were taken after 3 weeks of incubation. Bar = 200 µm. (G) Endocytosis phenotype of the wild‐type PH‐1 and various complementation strains. Mycelia were stained with FM4‐64 for 10 min before imaging. Bar = 10 µm.

Sequence analysis indicated that FgCapA contains two conserved F‐actin‐capping motifs and FgCapB contains one F‐actin‐capping motif (Fig. 7A). To analyse the role of the F‐actin‐capping motifs in FgCapA and FgCapB, we constructed the Δ*FgcapA*‐C‐ΔA1, Δ*FgcapA*‐C‐ΔA2 and Δ*FgcapB*‐C‐ΔB strains. The native promoter and ORF of *FgCAPA* lacking the F‐actin‐capping A1 motif was fused with a GFP fragment, and the resulting cassette was transformed into a Δ*FgcapA* strain background. The resulting transformant was identified and designated as Δ*FgcapA*‐C‐ΔA1. Using a similar procedure, Δ*FgcapA*‐C‐ΔA2 expressing *FgCAPA‐GFP* and lacking the F‐actin‐capping A2 motif in the Δ*FgcapA* strain and Δ*FgcapB*‐C‐ΔB expressing *FgCAPB‐GFP* and lacking the F‐actin‐capping B motif in the Δ*FgcapB* strain were generated.

As shown in Fig. 7B, Δ*FgcapA*‐C‐ΔA1 and Δ*FgcapA*‐C‐ΔA2 failed to complement the growth defects of Δ*FgcapA*, indicating that both motifs A1 and A2 are important for the function of FgCapA on vegetative growth. In these two strains, the GFP signals diffused evenly in the cytoplasm of mycelia without an actin localization pattern (Fig. 7C). Further investigation indicated that the complementation strains lacking motif A1 or A2 showed similar defects in conidiation, sexual reproduction and endocytosis to those of Δ*FgcapA* strain (Fig. 7D–G). However, FgCapB‐GFP lacking F‐actin‐capping motif B still exhibited actin localization in the Δ*FgcapB*‐C‐ΔB strain (Fig. 7C). Moreover, the Δ*FgcapB*‐C‐ΔB strain demonstrated similar mycelial growth rates and all tested phenotypes to those of *ΔFgcapB*‐C and the wild‐type strain PH‐1 (Fig. 7B,D–G).

**Figure 8:**
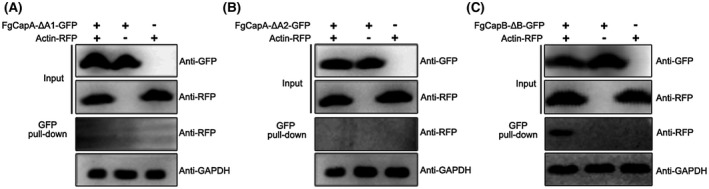
F‐actin‐capping motifs are indispensable for the interaction between FgCapA and actin. The interaction between Δ*FgcapA*‐C‐ΔA1 (A), Δ*FgcapA*‐C‐ΔA2 (B) or Δ*FgcapA*‐C‐ΔB (C) and actin‐RFP was examined by the co‐immunoprecipitation (Co‐IP) assay. Total protein (Input) extracted from the strain bearing FgCap‐GFP lacking individual F‐actin‐capping motif and actin‐RFP constructs or a single construct (FgCap‐GFP lacking individual F‐actin‐capping motif or Actin‐RFP) were subjected to SDS‐PAGE, and immunoblots were incubated with anti‐GFP and anti‐RFP antibodies, as indicated (Input panel). Each protein sample was pulled down using anti‐GFP agarose and further detected with anti‐RFP antibody (GFP pull‐down panel). Protein samples were also detected with anti‐GAPDH antibody as a reference.

To further verify whether the F‐actin‐capping motifs are essential for interaction between CAPs and actin *in vivo*, the actin‐RFP fusion construct was individually introduced into the Δ*FgcapA*‐C‐ΔA1, Δ*FgcapA*‐C‐ΔA2 and Δ*FgcapB‐*C‐ΔB. Total protein was isolated from the positive transformants and Co‐IP analysis was performed. As shown in Fig. 8A,B, FgCapA lacking either the F‐actin‐capping A1 or A2 motif was unable to interact with actin protein. In consistence with phenotypes of Δ*FgcapB*‐C‐ΔB, FgCapB lacking F‐actin‐capping B motif did not affect the interaction between FgCapB and actin (Fig. 8C). Taken together, these results indicated that the F‐actin‐capping motifs were essential for the functions of FgCapA but not for FgCapB in *F. graminearum*.

### FgCAP deletion mutants have attenuated DON production and virulence

FgCapA and FgCapB interacted with Tri1 and FgMyo1 and were localized partially to the toxisome (Figs 1 and S2). Additionally, both *FgCAPA* and *FgCAPB* showed relatively high transcriptional levels in the DON‐inducing TBI medium and during the infection process *in planta* (Fig. 4A). Thus, we were interested in investigating the function of FgCapA and FgCapB in toxisome formation, DON production and virulence.

**Figure 9:**
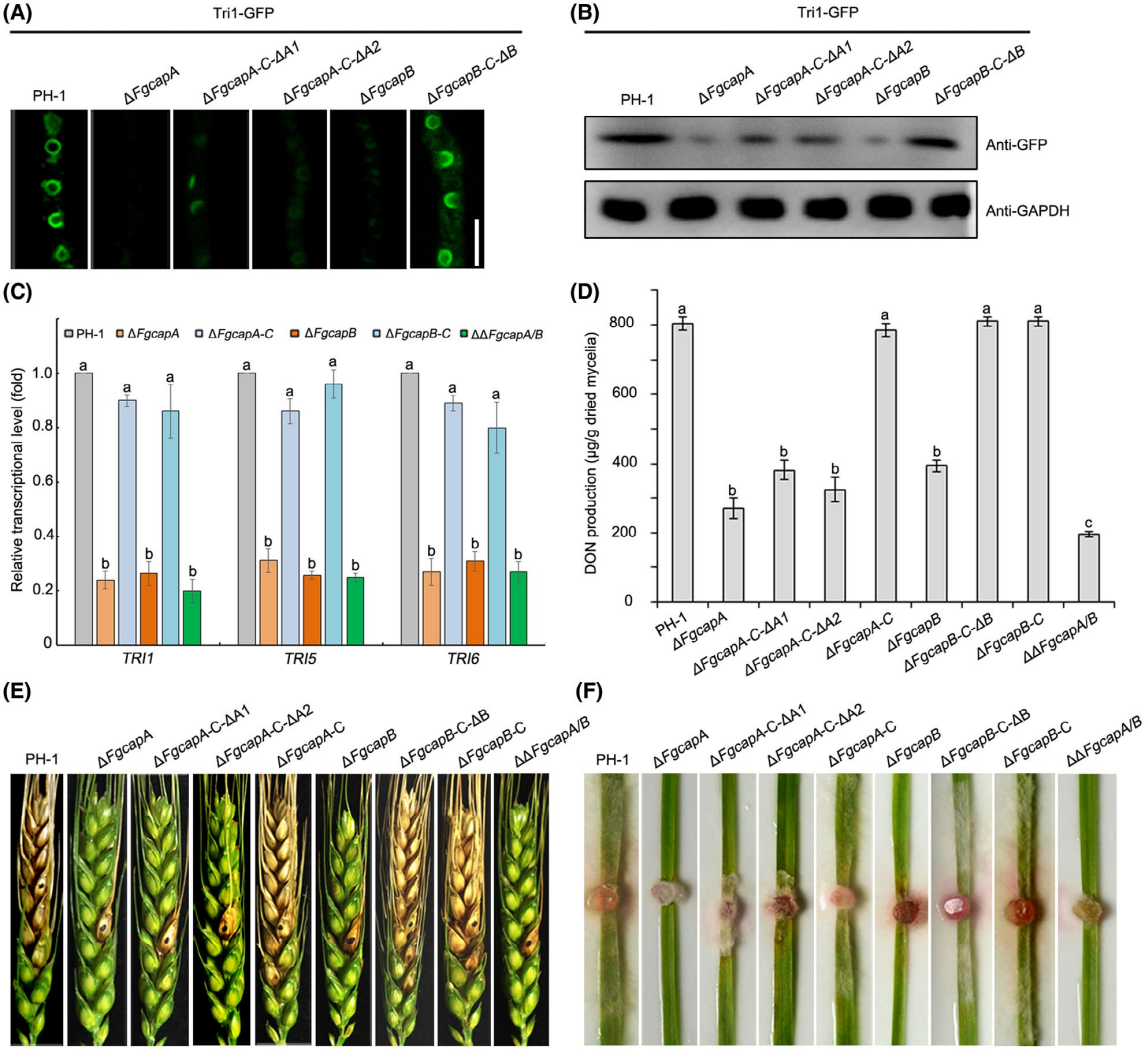
Capping gene deletion mutants attenuated toxisome formation, DON production and virulence *in planta*. (A) Toxisome formation in *Fusarium graminearum* wild‐type PH‐1, Δ*FgcapA*, Δ*FgcapB* and F‐actin‐capping motif truncated mutants. All strains were labelled with Tri1‐GFP as a toxisome indicator and incubated in trichothecene biosynthesis induction (TBI) medium for 48 h before imaging. Bar = 10 µm. (B) The accumulation of the Tri1‐GFP protein in each strain was further determined by western blot assay using the anti‐GFP antibody. The protein samples were also detected with anti‐GAPDH antibody as a reference. (C) Relative mRNA expression level of *TRI1*, *TRI5* and *TRI6* in the strains tested. After culturing in TBI for 2 days, mycelia of each strain were harvested for mRNA extraction. The *Actin* was used as a reference gene. (D) DON production in the wild‐type, mutant and various complementation strains after 7 days of incubation in TBI. Bars denote standard deviations from three experiments. Columns labelled with the same letter are not significantly different according to the LSD test at *P* = 0.05. (E) Virulence of the wild‐type, mutant and various complementation strains on wheat heads. Infected wheat heads were examined at 15 days after inoculation with a conidial suspension of each strain. The inoculation sites are indicated as black dots. (F) Disease symptoms on young wheat leaves infected by the wild‐type, mutant and various complementation strains. The images were taken at 6 days post‐inoculation.

As described previously, Tri1‐GFP can be used as an indicator for DON‐toxisome formation (Tang *et al.*, 2018), thus Tri1‐GFP was transformed into the wild‐type and CAP mutant strains. As shown in Fig. 9A, expression of Tri1‐GFP was highly induced, and typical spherical toxisomes were formed in the mycelia of the wild‐type strain after 2 days of incubation in TBI. In contrast, the Tri1‐GFP signals in Δ*FgcapA* and Δ*FgcapB* mutants dramatically decreased, and very faint toxisomes were observed (Fig. 9A). Western blot analysis confirmed that the amounts of the Tri1‐GFP protein in the CAP mutants were also considerably lower than that in the wild‐type strain (Fig. 9B). Meanwhile, the transcriptional levels of the *TRI* genes in the deletion mutants were assayed by RT‐qPCR after incubation in TBI medium. Three selected *TRI* genes (*TRI1*, *TRI5* and *TRI6*) were dramatically down‐regulated in the Δ*FgcapA* and Δ*FgcapB* mutant, compared to those in the wild‐type (Fig. 9C). Consistent with toxisome formation and expression of *TRI* genes, CAP mutants produced less DON than the wild‐type strain after 7 days of incubation in TBI (Fig. 9D). These results suggest that FgCaps participated in expression of *TRI* gene and toxisome formation, and were important for DON biosynthesis in *F. graminearum*.

DON is a key virulence factor in *F. graminearum*, thus the virulence of the CAP mutants was evaluated by point‐inoculating conidial suspensions in flowering wheat heads and mycelial plugs on the leaves of wheat seedlings. On wheat heads inoculated with the wild‐type strain PH‐1, scab symptoms first developed on the inoculated spikelets and rapidly spread to the neighbouring wheat head. Fifteen days after inoculation, severe and typical scab symptoms were caused by the wild‐type and complementation strains. In contrast, scab symptoms caused by mutants were restricted to the inoculated spikelets and failed to spread from the inoculated floret to the rachis (Fig. 9E). Additionally, all mutants demonstrated attenuated virulence on seedling leaves (Fig. 9F). The complementation strains Δ*FgcapA*‐C and Δ*FgcapB*‐C exhibited wild‐type levels of DON production and virulence (Fig. 9D–F). Moreover, F‐actin‐capping motif truncated mutant Δ*FgcapA*‐C‐ΔA1 and Δ*FgcapA*‐C‐ΔA2 demonstrated similar reduction of DON production and virulence as Δ*FgcapA* mutant. The pathogenicity and DON biosynthesis of Δ*FgcapB‐*C‐ΔB mutant was the same as that of wild type, as other tested phenotypes (Fig. 9C–F). Collectively, our results suggest that CAPs are important for toxisome formation, DON production and virulence in *F. graminearum*.

## Discussion

The actin cytoskeleton plays an important yet poorly understood role during cellular development in eukaryotes. Remodelling of the actin cytoskeleton in response to internal signals or environmental stimuli is controlled by a plethora of actin‐binding proteins, including the CAP proteins (Cooper and Sept, 2008). Although CAPs have been investigated in yeast, *Arabidopsis* and mammalian cells (Amatruda *et al.*, 1990; Huang *et al.*, 2003; Kim *et al.*, 2004; Sinnar *et al.*, 2014), the function of CAPs in filamentous fungi is still largely unknown. Here, we identified and genetically characterized two CAPs, FgCapA and FgCapB, in the plant pathogenic fungus *F. graminearum*. FgCapA interacted with FgCapB. Deletion of *FgCAPA* and *FgCAPB* resulted in various defects, including a reduction in hyphal growth and conidiation, sexual reproduction and decreased endocytosis, DON biosynthesis and virulence in *F. graminearum* (Figs 4, 5, 6, 7 and 9). The double gene deletion mutant exhibited similar phenotypes to the single gene deletion mutants, supporting the hypothesis that the two CAP proteins form a heterodimer. These results were consistent with studies of other eukaryotes (Amatruda *et al.*, 1990; Blanchoin *et al.*, 2014; Li *et al.*, 2017b). In yeast and *M. oryzae,* the F‐actin‐capping motifs of CapA were required to interact with actin (Amatruda *et al.*, 1990; Li *et al.*, 2017b). Similarly, in the current study, we found that FgCapA interacted with actin, and the interaction depended on its F‐actin‐capping motifs (Figs 3 and 8). Unexpectedly, although FgCapB also interacted with actin, Δ*FgcapB*‐C‐ΔB completely rescued the defects of the Δ*FgcapB* mutant, indicating that the F‐actin‐capping motif B was not required for the function of FgCapB, which was different from the results of previous studies in other fungi (Amatruda *et al.*, 1990; Li *et al.*, 2017b). Other unidentified motif(s) in FgCapB may be responsible for its interaction with actin. Taken together, these results indicate that CAPs may have species‐specific interaction patterns with actin in different organisms.

The DON biosynthetic organelle, the toxisome, is a remodelled perinuclear endoplasmic reticulum in *F. graminearum* (Boenisch *et al.*, 2017, 2019; Flynn *et al.*, 2019; Tang *et al.*, 2018). The assembly of toxisomes is mediated by the myosin‐actin cytoskeleton (Tang *et al.*, 2018). Inactivation of FgMyo1 by the fungicide phenamacril or disruption of F‐actin formation using latrunculin A dramatically inhibited toxisome formation and DON biosynthesis (Tang *et al.*, 2018; Zhang *et al.*, 2015; Zheng *et al.*, 2015). Additionally, deletion of the actin‐associated protein genes *FgPRK1* or *FgEND3* also hindered toxisome formation and reduced the concentration of trichothecenes that accumulate in cultures (Tang *et al.*, 2018). In this study, we further found that actin CAPs interact with actin (Fig. 3) and the deletion of *FgCAPA or FgCAPB* leads to defects in toxisome formation and subsequent DON production (Fig. 9), indicating that FgCaps are required for toxisome formation. Moreover, previous studies have shown that the trichothecene efflux pump Tri12 interacts with toxisomes and may participate in DON transport through vesicles and vacuoles. The motility of vesicles containing Tri12 was largely dependent on filamentous actin (Menke *et al.*, 2012, 2013; Roze *et al.*, 2011), suggesting that the actin cytoskeleton may be involved not only in toxisome formation but also in DON export. Thus, it is likely that the actin cytoskeleton can be used as a potential target for the development of new fungicides to inhibit DON biosynthesis.

Emerging evidence has demonstrated that the actin cytoskeleton plays an important role during plant–microbe interactions. In *Arabidopsis* epidermal cells, cortical actin was remodelled within minutes for defence responses in response to pathogenic and nonpathogenic microbes and diverse microbe‐associated molecular patterns (MAMPs) (Henty‐Ridilla *et al.*, 2013, 2014). Capping proteins integrate multiple MAMP signalling pathways to modulate actin dynamics for plant resistance against bacterial and fungal phytopathogens (Li *et al.*, 2012, 2015, 2017a). Moreover, the actin cytoskeleton is critical for virulence in plant pathogens. For example, assembly of an F‐actin network was required for initiation of the infection process in *M. oryzae* (Kankanala *et al.*, 2007). CAP mutants in *M. oryzae* dramatically reduced fungal virulence *in planta* (Li *et al.*, 2017b). In *Botrytis cinerea*, deletion of the capping gene *BcCAP1* impaired the ability of the pathogen to penetrate host tissue and its subsequent invasive growth in the leaves of *Phaseolus vulgaris* (Gonzalez‐Rodriguez *et al.*, 2016). Here, we found that the deletion mutants of CAP genes attenuate the disease symptoms, although they could successfully penetrate the hosts, indicating that CAPs play an important role in *F. graminearum* growth during invasion (Fig. 9E,F). DON has been well characterized as a critical virulence factor for the spread of *F. graminearum* within wheat spikelets (Desjardins *et al.*, 1996), therefore the reduced spread of the capping gene mutants during invasion could primarily result from the defect in DON production (Fig. 9D). Additionally, the growth defect of the mutants may affect the development of disease symptoms *in planta* (Fig. 4B). Taken together, the defects of hyphal growth and DON biosynthesis contributed to the attenuated virulence of the *F. graminearum* actin capping mutants on the host plants.

## Experimental Procedures

### Fungal strains and culture conditions

As a parental strain, the *F. graminearum* strain PH‐1 was used in this study. The wild‐type and mutant strains were grown at 25 °C on potato dextrose agar (PDA) (200 g potato, 20 g glucose, 10 g agar and 1 L water), minimal medium (MM) (10 mM K_2_HPO_4_, 10 mM KH_2_PO_4_, 4 mM (NH_4_)_2_SO_4_, 2.5 mM NaCl, 2 mM MgSO_4_, 0.45 mM CaCl_2_, 9 mM FeSO_4_, 10 mM glucose, 1% agar and 1 L water, pH 6.9) and complete medium (CM) (1% glucose, 0.2% peptone, 0.1% yeast extract, 0.1% casamino acids, nitrate salts, trace elements, 0.01% vitamins and 1 L water, pH 6.5) to determine the growth rate and colony morphology.

For conidiation assays, ten mycelial plugs (5 mm in diameter) of each strain were taken from the periphery of a 3‐day‐old colony and inoculated in a 50‐mL flask containing 20 mL of CMC liquid medium (15 g carboxylmethyl cellulose, 1 g yeast extract, 0.5 g MgSO_4_, 1 g NH_4_NO_3_, 1 g KH_2_PO_4_ and 1 L water) (Cappellini and Peterson, 1965). The flasks were incubated at 25 °C for 4 days in a shaker (180 rpm), and the resulting conidia were germinated in distilled water with 2% sucrose. Perithecium formation was assayed on carrot agar medium (CA) (200 g carrot, 20 g agar and 1 L water) at 25 °C under a 12/12 h light/dark cycle. Mycelia were grown on CA for 7 days and then rubbed with a glass spreader after applying 0.1% sterilized Tween 20 solution to induce sexual reproduction. Liquid trichothecene biosynthesis induction (TBI) medium (30 g sucrose, 1 g KH_2_PO_4_, 0.5 g MgSO_4_.7 H_2_O, 0.5 g KCl, 0.01 g FeSO_4_.7H_2_O, 1.47 g putrescine hydrochloride, trace elements and 1 L water, pH 4.5) were used for toxisome formation and DON production (Gardiner *et al*., 2009; Menke *et al.*, 2012). Strains were grown at 28 °C in the dark for 2 days before toxisome observation.

The sensitivity of the strains to stress agents was determined as described previously (Liu *et al.*, 2015). The final concentrations of SDS, H_2_O_2_ and Congo red in PDA are indicated in the figure. The mycelial growth inhibition rate (MGIR) was calculated using the formula MGIR% = [(*N* – *C*)*/C*] × 100, where *C* is the colony diameter of the control without treatment and *N* is that with treatment. Each experiment was repeated three times independently.

### Construction of gene deletion mutants and complementation strains

The deletion mutants Δ*FgcapA* and Δ*FgcapB* were generated using a previously described protocol (Yun *et al*., 2013). To generate the double mutant of *FgCAPA* and *FgCAPB*, *FgCAPA* was knocked out in the Δ*FgcapB* mutant and the resulting double mutant was designated ΔΔ*FgcapA/B*. The primers used to amplify the flanking sequences for each gene are listed in Table S1. Deletion mutants were identified by PCR with relevant primers and a Southern blot assay (Fig. S3).

To construct the *FgCAPA‐GFP* fusion cassette, the *FgCAPA* fragment containing the native promoter and ORF (without the stop codon) was amplified with primers P17 and P18 (Table S1). The resulting PCR products were co‐transformed with *Xho*I‐digested pYF11‐GFP plasmid into *S. cerevisiae* XK1‐25 using the alkali‐cation yeast transformation kit (MP Biomedicals, Solon, USA). The recombined pYF11‐*FgCAPA‐GFP* plasmid was recovered from the yeast transformant using a yeast plasmid extraction kit (Solarbio, Beijing, China) and then transferred into *Escherichia coli* strain DH5α for amplification. The recombinant plasmid pYF11‐*FgCAPA‐GFP* was transformed into the Δ*FgcapA* mutant for complementation, and the resulting transformant was designated Δ*FgcapA*‐C. Using the same strategy, the pYF11‐*FgCAPB‐GFP* recombinant plasmid was constructed and transformed into the Δ*FgcapB* mutant and generated the complementation Δ*FgcapB*‐C.

### Microscopic examinations

For toxisome observation, each strain labelled with Tri1‐GFP was grown in liquid TBI medium at 28 °C in darkness for 2 days prior to examination under a Zeiss LSM780 confocal microscope (Gottingen, Niedersachsen, Germany). The following confocal microscopy settings were used for GFP observation: laser 488 nm at 50% power, pinhole 90 μm, master gain 580. Conidial germination was stained with CFW at a concentration of 0.1 mg/mL for 30 s before confocal observation. To observe endocytosis, fresh conidia or mycelia were collected and strained with FM4‐64 at a concentration of 7.5 μM for 1, 5 or 10 min in the dark at room temperature (Li *et al.*, 2017b). The laser excitation wavelength was set at 561 nm for FM4‐64, mCherry and RFP (red fluorescence).

### Bimolecular fluorescence complementation assay

For BiFC strain construction, the ORF fragments of *TRI1* and *FgCAPA* were respectively fused into *Xho*I‐digested pHZ65 vector harbouring YFP^N^ and the hygromycin B resistance cassette, and pHZ68 vector that carries YFP^C^ and the zeocin resistance cassette. The final plasmid constructs of pYFP^N^‐Tri1 and pFgCapA‐YFP^C^ were verified by sequencing and then co‐transformed into the protoplasts of PH‐1 in pairs. Transformants resistant to both hygromycin and zeocin were isolated and confirmed by PCR. The recombination plasmid pYFP^N^‐Tri1 or pFgCapA‐YFP^C^ was individually transformed into PH‐1, and resultant transformants were used as negative controls. YFP signals in the mycelia grown in TBI for 48 h were examined under a confocal microscope.

### Western blot assay

Protein isolation was performed as previously described (Yun *et al.*, 2015). The resulting proteins were separated by 10% SDS polyacrylamide gel electrophoresis (SDS‐PAGE) and transferred onto an Immobilon‐P transfer membrane (Millipore, Billerica, MA, USA). Monoclonal anti‐GFP (ab32146, Abcam, Cambridge, UK), anti‐mCherry (ab125096, Sigma, St Louis, MO, USA), anti‐RFP (ab65856, Sigma) and anti‐Flag (A9044, Sigma) antibodies were used at a 1:10 000 dilution for immunoblot analyses. The samples were also detected with monoclonal anti‐GAPDH antibody (EM1101, HuaAn Biotechnology Co., Ltd, Hangzhou, Zhejiang, China) as a control.

### Coimmunoprecipitation assay

The GFP, RFP, 3 × Flag or mCherry fusion constructs were verified by DNA sequencing and transformed in pairs into PH‐1. Transformants expressing pairs of fusion constructs were confirmed by western blot analysis. In addition, the transformants expressing a single fusion construct were used as references. For Co‐IP assays, total proteins were extracted and incubated with anti‐GFP or anti‐Flag agarose. Proteins eluted from agarose were analysed by western blot detection with a polyclonal anti‐Flag or an anti‐GFP antibody. The protein samples were also detected with monoclonal anti‐GAPDH antibody as a reference. Each experiment was repeated twice.

### Yeast two‐hybrid assay

To construct plasmids for the yeast two‐hybrid analysis, the ORFs of the *FgCAPA* and *FgCAPB* genes were amplified using PH‐1 cDNA as a template. The PCR product was inserted into the yeast GAL4‐binding domain vector pGBKT7 or GAL4 activation domain vector pGADT7 (Clontech, Mountain View, CA, USA). The pairs of yeast two‐hybrid plasmids were co‐transformed into the *S. cerevisiae* strain AH109 following the LiAc/ss‐DNA/PEG (lithium acetate/single‐stranded DNA/polyethylene glycol) transformation protocol (Yu *et al*., 2014). In addition, the plasmids pGBKT7‐53 and pGADT7 served as positive controls, while the plasmids pGBKT7‐Lam and pGADT7 served as negative controls. Transformants were grown at 30 °C for 3 days on synthetic medium (SD) lacking Leu and Trp and then transferred to SD lacking His, Leu, Trp and Ade to assess interaction. Three independent experiments were performed to confirm the results.

### Plant infection, *TRI* gene expression and DON production assays

The conidia of each strain formed in CMC medium were collected and suspended in sterile distilled water to a final concentration of 10^5^ conidia/mL. A 10 μL suspension of fresh conidia of each strain was injected into a floret in the central section spikelet of single flowering wheat head of the susceptible cultivar Zimai22. At 15 days after inoculation, the infected spikelets in each inoculated wheat head were recorded. For wheat leaf infection, fresh mycelial plugs of each strain were inoculated in the middle of the leaves and incubated in a growth chamber at 25 °C. Images were taken 6 days after inoculation. There were 15 replicates for each strain in each experiment, and these experiments were repeated three times.

To compare the *TRI* genes expression, the wild‐type and CAP mutant strains were inoculated into TBI medium and cultured at 28 °C in a shaker (150 rpm). After 2 days of incubation, mycelia of each sample were harvested and the total RNA was extracted. The mRNA expression of *TRI1*, *TRI5* and *TRI6* in PH‐1 and the mutants was determined using a quantitative reverse transcription PCR (RT‐qPCR) method as described previously (Liu *et al.*, 2015). The experiment was repeated three times. To quantify DON production, each strain was grown shaking (150 rpm) in TBI medium at 28 °C for 7 days in darkness. The supernatant was collected by filtration through three layers of gauze and then purified and quantified using the LC‐MS/MS system (Tang *et al.*, 2018). The experiment was repeated three times.

## Supporting information


**Fig. S1** Phylogenetic analysis of the putative CAPs from *Fusarium graminearum*, two yeasts and five filamentous fungi. Amino acid sequences of CapA and CapB orthologues were aligned using CLUSTALW, and a neighbour‐joining tree was generated by MEGA 5.0. The names or loci of proteins are indicated in the figure.

**Fig. S2** FgCapB interacts with FgMyo1 and Tri1. (A) The interaction of FgCapB‐GFP and FgMyo1‐Flag was verified by the co‐immunoprecipitation (Co‐IP) assay. Total protein (Input) extracted from the strain bearing FgCapB‐GFP and FgMyo1‐Flag constructs or a single construct (FgCapB‐GFP or FgMyo1‐Flag) were subjected to SDS‐PAGE and immunoblots were incubated with anti‐FLAG and anti‐GFP antibodies, as indicated (Input panel). Each protein sample was pulled down using anti‐Flag agarose and further detected with anti‐GFP antibody (Flag pull‐down panel). Protein samples were also detected with anti‐GAPDH antibody as a reference. (B) The interaction of FgCapB‐mCherry and Tri1‐GFP was verified by the Co‐IP assay. Protein samples were pulled down using anti‐GFP agarose and further detected with an anti‐mCherry antibody. Protein samples were also detected with anti‐GAPDH antibody as a reference. (C) FgCapB‐mCherry was partially colocalized with Tri1‐GFP on DON‐toxisomes at 48 h of incubation in trichothecene biosynthesis induction (TBI) medium. Bar = 10 µm.

**Fig** **.** **S3** Identification of deletion mutants. (A) PCR identification of deletion mutants Δ*FgcapA* and Δ*FgcapB*. (B) Southern blot analysis of the deletion mutants of Δ*FgcapA* and Δ*FgcapB *using the hygromycin fragment as the probe. Δ*FgcapA *had an anticipated 4539 bp band, but lacked the 4539 bp band presented in *Fusarium graminearum* wild‐type PH‐1. Δ*FgcapB* had an anticipated 5172 bp band, but lacked the 5172 bp band presented in the wild‐type PH‐1.

**Fig. S4** Deletion mutants of* FgCAP* genes reduced the rate of hyphal growth and altered the morphologies of conidia. (A) Colony diameter of *Fusarium graminearum* wild‐type PH‐1, Δ*FgcapA*, Δ*FgcapB* and ΔΔ*FgcapA/B *grown on potato dextrose agar (PDA), complete medium (CM) and minimal medium (MM) agar plates for 3 days at 25 °C. Bars denote standard deviations from three experiments. Columns labelled with the same letter are not significantly different according to the least significant difference (LSD) test at *P* = 0.05. (B) Ratio of the different number of conidial septa in PH‐1, mutants and complemented strains harvested from 4‐day‐old carboxymethyl cellulose (CMC) cultures. (C) The representative conidial morphology of the wild‐type PH‐1 and mutants. The septa were stained with calcofluor white and imaged with a fluorescence microscope. Bar = 20 µm.

**Table S1** A list of primers used in this study.
