# Supplementary figures and images for: Capping proteins regulate fungal development, DON‐toxisome formation and virulence in Fusarium graminearum
Source: Mol Plant Pathol. 2019 Nov 6;21(2):173–87. doi: 10.1111/mpp.12887 (PMC6988429; doi:10.1111/mpp.12887)

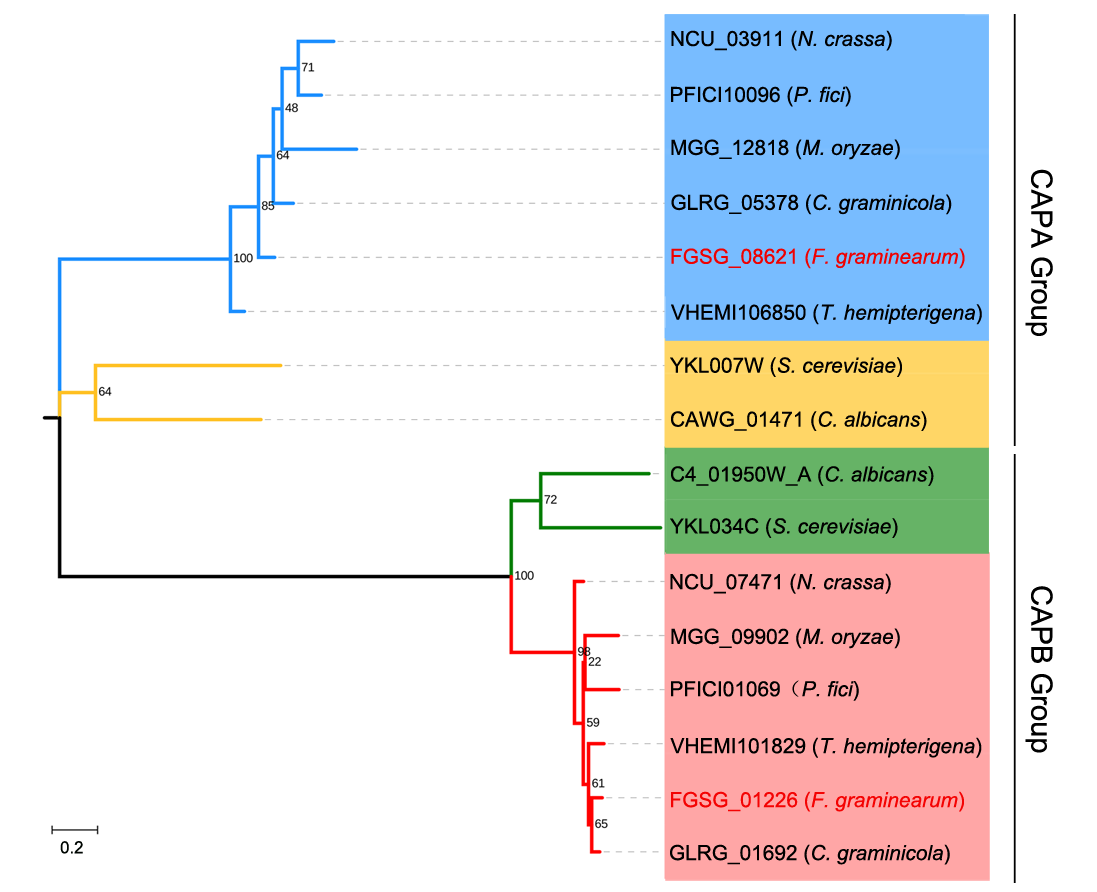

Supplement: Supplementary file 1 — Fig. S1 Phylogenetic analysis of the putative CAPs from Fusarium graminearum, two yeasts and five filamentous fungi. Amino acid sequences of CapA and CapB orthologues were aligned using CLUSTALW, and a neighbour‐joining tree was generated by MEGA 5.0. The names or loci of proteins are indicated in the figure. [file MPP-21-173-s001.tif]

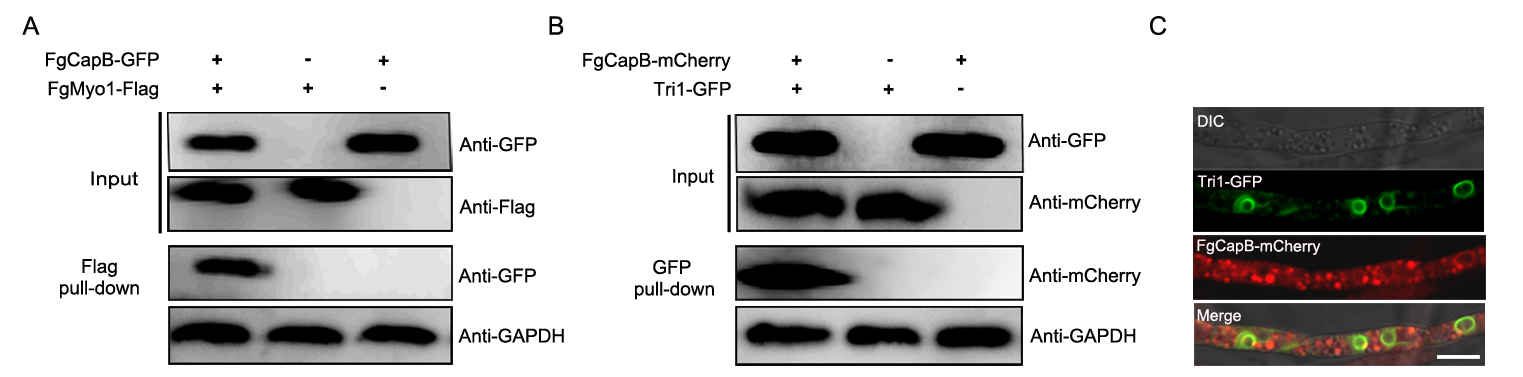

Supplement: Supplementary file 2 — Fig. S2 FgCapB interacts with FgMyo1 and Tri1. (A) The interaction of FgCapB‐GFP and FgMyo1‐Flag was verified by the co‐immunoprecipitation (Co‐IP) assay. Total protein (Input) extracted from the strain bearing FgCapB‐GFP and FgMyo1‐Flag constructs or a single construct (FgCapB‐GFP or FgMyo1‐Flag) were subjected to SDS‐PAGE and immunoblots were incubated with anti‐FLAG and anti‐GFP antibodies, as indicated (Input panel). Each protein sample was pulled down using anti‐Flag agarose and further detected with anti‐GFP antibody (Flag pull‐down panel). Protein samples were also detected with anti‐GAPDH antibody as a reference. (B) The interaction of FgCapB‐mCherry and Tri1‐GFP was verified by the Co‐IP assay. Protein samples were pulled down using anti‐GFP agarose and further detected with an anti‐mCherry antibody. Protein samples were also detected with anti‐GAPDH antibody as a reference. (C) FgCapB‐mCherry was partially colocalized with Tri1‐GFP on DON‐toxisomes at 48 h of incubation in trichothecene biosynthesis induction (TBI) medium. Bar = 10 µm. [file MPP-21-173-s002.tif]

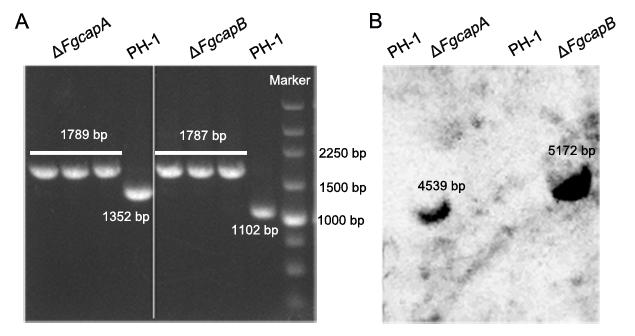

Supplement: Supplementary file 3 — Fig . S3 Identification of deletion mutants. (A) PCR identification of deletion mutants ΔFgcapA and ΔFgcapB. (B) Southern blot analysis of the deletion mutants of ΔFgcapA and ΔFgcapB using the hygromycin fragment as the probe. ΔFgcapA had an anticipated 4539 bp band, but lacked the 4539 bp band presented in Fusarium graminearum wild‐type PH‐1. ΔFgcapB had an anticipated 5172 bp band, but lacked the 5172 bp band presented in the wild‐type PH‐1. [file MPP-21-173-s003.tif]

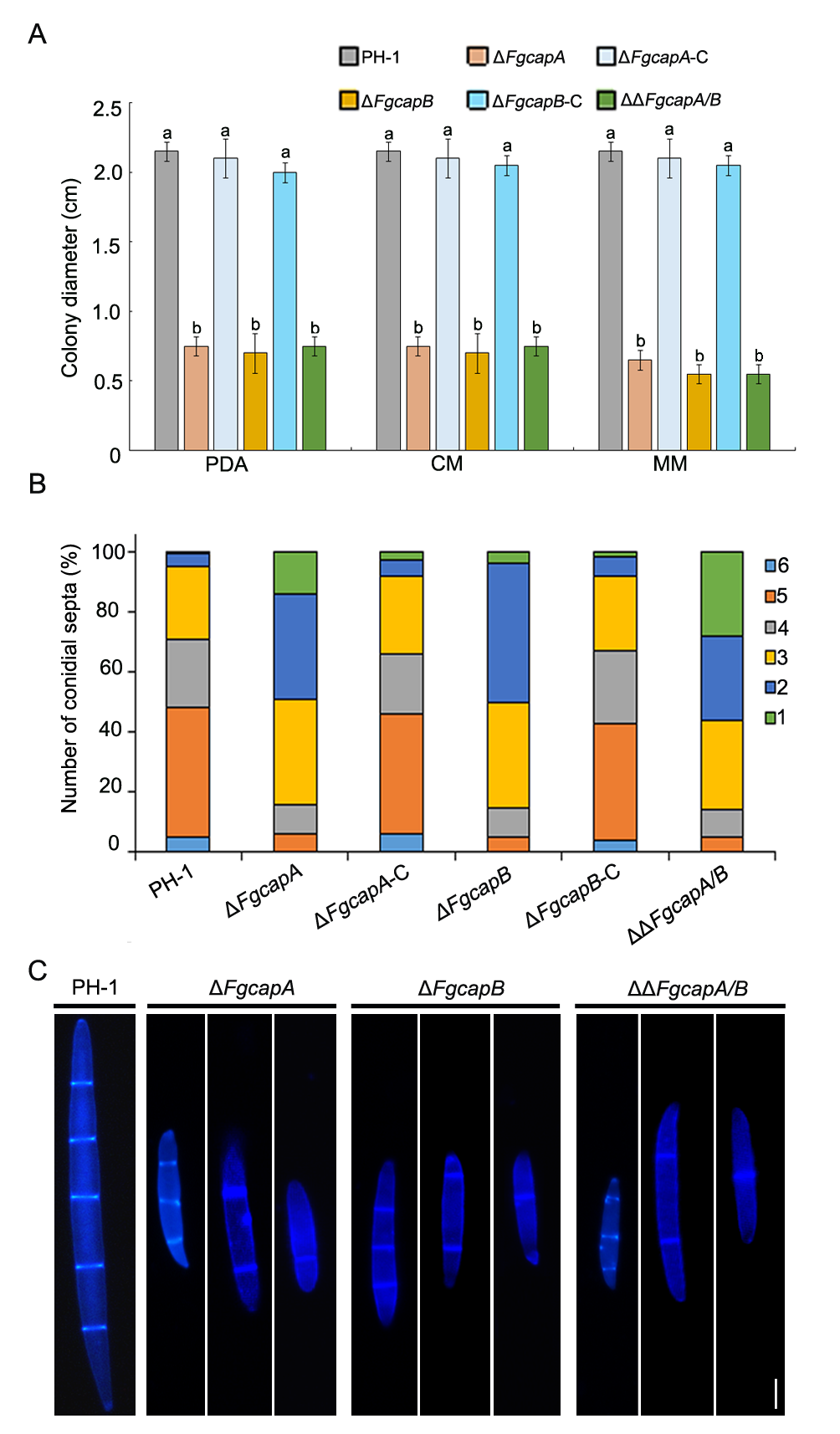

Supplement: Supplementary file 4 — Fig. S4 Deletion mutants of FgCAP genes reduced the rate of hyphal growth and altered the morphologies of conidia. (A) Colony diameter of Fusarium graminearum wild‐type PH‐1, ΔFgcapA, ΔFgcapB and ΔΔFgcapA/B grown on potato dextrose agar (PDA), complete medium (CM) and minimal medium (MM) agar plates for 3 days at 25 °C. Bars denote standard deviations from three experiments. Columns labelled with the same letter are not significantly different according to the least significant difference (LSD) test at P = 0.05. (B) Ratio of the different number of conidial septa in PH‐1, mutants and complemented strains harvested from 4‐day‐old carboxymethyl cellulose (CMC) cultures. (C) The representative conidial morphology of the wild‐type PH‐1 and mutants. The septa were stained with calcofluor white and imaged with a fluorescence microscope. Bar = 20 µm. [file MPP-21-173-s004.tif]
