# Supplementary material for: Capping proteins regulate fungal development, DON‐toxisome formation and virulence in Fusarium graminearum
Source: Mol Plant Pathol. 2019 Nov 6;21(2):173–87. doi: 10.1111/mpp.12887 (PMC6988429; doi:10.1111/mpp.12887)
Supplement: Supplementary file 5 — Table S1 A list of primers used in this study. [file MPP-21-173-s005.doc]

**Table S1. A list of primers used in this study**

| Code | Name | Sequence (5'to 3') | Products |
| --- | --- | --- | --- |
| 1 | FgCapA-P1 | CGTAGCAACGATCCCTGCGTCT | Upstream fragment of FgCapA |
| 2 | FgCapA-P2 | CAAAATAGGCATTGATGTGTTGACCTCCTGTAAATTGGGAGCTTAGCTT |
| 3 | FgCapA-P3 | CTCGTCCGAGGGCAAAGGAATAGAGTAGCCTGTCACCACTAAAGCTAAT | Downstream fragment of FgCapA |
| 4 | FgCapA-P4 | GATGTGGTTCATCTGTCATGT |
| 5 | FgCapA-ID-F | CTGTAAAATGCATGAAGACA | Identification FgCapA deletion transformants |
| 6 | FgCapA-ID-R | TGTCGTCGCAGACCCTTGAATG |
| 7 | FgCapB-P1 | GCTTACTGTCTGATATGTGGC | Upstream fragment of FgCapB |
| 8 | FgCapB-P2 | CAAAATAGGCATTGATGTGTTGACCTCCGCGGTATATCGGGTTCTCTGG |
| 9 | FgCapB-P3 | CTCGTCCGAGGGCAAAGGAATAGAGTAGGAGGAAGAAGCACCCACTAT | Downstream fragment of FgCapB |
| 10 | FgCapB-P4 | GCCACCACCTGTCGTGAAGT |
| 11 | FgCapB-ID-F | ATGGCTTTGTCGTCAAGTCC | Identification FgCapB deletion transformants |
| 12 | FgCapB-ID-R | AGTAGATTGCGCATCTTGAGT |
| 13 | HPH-F | GGAGGTCAACACATCAATGCCTATT | Fragment of HPH (Hygromycin B) resistance gene |
| 14 | HPH-R | CTACTCTATTCCTTTGCCCT |
| 15 | NEO-F | GGAGGTCAACACATCAATGCT | Fragment of Neo (Neomycin) resistance gene |
| 16 | NEO-R | TCAGAAGAACTCGTCAAGAAG |
| 17 | FgCapA-GFP-F | GACCTCGAGGGGGGGCCCCGTAGCAACGATCCCTGCGTCT | A pair of PCR primers to amplify CapA fragments used for construction of the FgCapA-GFP, vector under its own promoter |
| 18 | FgCapA-GFP-R | CTCCTCGCCCTTGCTCACCATTCGCCTCGAGCCTCCGTTGCC |
| 19 | FgCapB-GFP-F | GACCTCGAGGGGGGGCCCGCTTACTGTCTGATATGTGGC | A pair of PCR primers to amplify CapB fragments used for construction of the FgCapB-RFP, vector under its own promoter |
| 20 | FgCapB-GFP-R | CTCCTCGCCCTTGCTCACCATTTTCCTCATGCTTCCAATGAGTT |
| 21 | mCherry-F | GTGAGCAAGGGCGAGGAGGAT | A pair of PCR primers to amplify  CapA fragments used for construction of the FgCapA -mCherry, vector under its own promoter |
| 22 | mCherry-R | TTATCACTTGTACAGCTCGTCC |
| 23 | HPH-mCherry-F | GGCGGCATGGACGAGCTGTACAAGTGATAAGGAGGTCAACACATCAATGCCTATT |
| 24 | HPH-mCherry-R | CTACTCTATTCCTTTGCCCT |
| 25 | FgCapA-mcherry-F | CGTAGCAACGATCCCTGCGTCT |
| 26 | FgCapA-mcherry-R: | GGCCATGTTATCCTCCTCGCCCTTGCTCACTCGCCTCGAGCCTCCGTTGCC |
| 27 | FgCapB-mcherry-F | GCTTACTGTCTGATATGTGGC | A pair of PCR primers to amplify CapB fragments used for construction of the FgCapB -mCherry, vector under its own promoter |
| 28 | FgCapB-mcherry-R | GGCCATGTTATCCTCCTCGCCCTTGCTCACTTTCCTCATGCTTCCAATGAGTT |
| 29 | Actin-RFP-F | ACTCACTATAGGGCGAATTGGGTACTCAAATTGGTTGCCAGTCGGAATGACTGGGTGA | A pair of PCR primers to amplify Fg Actin fragments used for construction of the Actin-RFP, vector under its own promoter |
| 30 | Actin-RFP-R | CATGAACTCCTTGATGACGTCCTCGGAGGAGGCCATGAAGCACTTGCGGTGAACGA |
| 35 | Tri1-GFP-F | ACTCACTATAGGGCGAATTGGGTACTCAAATTGGTTTTGTGAGTAGGCCTCATA | A pair of PCR primers to amplify Tri1 fragments used for construction of the Tri1-GFP, vector under its own promoter |
| 36 | Tri1-GFP-R | CACCACCCCGGTGAACAGCTCCTCGCCCTTGCTCACGTCATCCTGTACCAATTCCAATCG |
| 37 | AD-CapA-F | GCCATGGAGGCCAGTGAATTCATGTCCGATATCGAAACCGTCT | Construction of pGADT7-FgCapA construct |
| 38 | AD-CapA-R | ATGCCCACCCGGGTGGAATTCCTATCGCCTCGAGCCTCCG |
| 39 | BD-CapA-F | ATGGCCATGGAGGCCGAATTCATGTCCGATATCGAAACCGTCT | Construction of pGBKT7-FgCapA construct |
| 40 | BD-CapA-R | TCGACGGATCCCCGGGAATTCCTATCGCCTCGAGCCTCCG |
| 41 | AD-CapB-F | GCCATGGAGGCCAGTGAATTCATGGCCGTCGATCCCTTTG | Construction of pGADT7-FgCapB construct |
| 42 | AD-CapB-R | ATGCCCACCCGGGTGGAATTCTCATTTCCTCATGCTTCCAATG |
| 43 | BD-CapB-F | ATGGCCATGGAGGCCGAATTCATGGCCGTCGATCCCTTTG | Construction of pGBKT7-FgCapB construct |
| 44 | BD-CapB-R | TCGACGGATCCCCGGGAATTCTCATTTCCTCATGCTTCCAATG |
| 45 | FgCapA-GFP-ΔA1-1-F | GACCTCGAGGGGGGGCCCCGTAGCAACGATCCCTGCGTCT | Construction of FgCapA-ΔA1-GFP |
| 46 | FgCapA-GFP-ΔA1-1-R | GTCAACCTTGAGAGAGCCC |
| 47 | FgCapA-GFP-ΔA1-2-F | GGGCTCTCTCAAGGTTGACCGTCTCCTGACCAACAAGCCTGTCTC |
| 48 | FgCapA-GFP-ΔA1-2-R | CTCCTCGCCCTTGCTCACCATTCGCCTCGAGCCTCCGTTGCC |
| 49 | FgCapA-GFP-ΔA2-1-F | GACCTCGAGGGGGGGCCCCGTAGCAACGATCCCTGCGTCT | Construction of FgCapA-ΔA2-GFP |
| 50 | FgCapA-GFP-ΔA2-1-R | GAATGCACCCTCGCTAAGGCTCAC |
| 51 | FgCapA-GFP-ΔA2-2-F | TCGTGAGCCTTAGCGAGGGTGCATTCCAGAAGATCGAATGGGACCG |
| 52 | FgCapA-GFP-ΔA2-2-R | CTCCTCGCCCTTGCTCACCATTCGCCTCGAGCCTCCGTTGCC |
| 53 | FgCapB-GFP-ΔB-1-F | GACCTCGAGGGGGGGCCCGCTTACTGTCTGATATGTGGC | Construction of FgCapB-ΔB-GFP |
| 54 | FgCapB-GFP-ΔB-1-R | GAGGAGGTAGTCTCGGCCGGTC |
| 55 | FgCapB-GFP-ΔB-2-F | GACCGGCCGAGACTACCTCCTCGGCGATAGCTACCGCTCACC |
| 56 | FgCapB-GFP-ΔB-2-R | CTCCTCGCCCTTGCTCACCATTTTCCTCATGCTTCCAATGAGTT |
| 57 | RT-Actin-F | ATCCACGTCACCACTTTCAA | qRT-PCR primers of *ACTIN* |
| 58 | RT-Actin-R | TGCTTGGAGATCCACATTTG |
| 59 | RT-TRI1-F | ACCACCCGCCAAACCCTC | qRT-PCR primers of *TRI1* |
| 60 | RT-TRI1-R | TTCAACAATGGGAGTGATTAG |
| 61 | RT-TRI5-F | AGCAGATGGTTGCTGTCTTCT | qRT-PCR primers of *TRI5* |
| 62 | RT-TRI5-R | TTCTGAGCCTCCTTCACATCG |
| 63 | RT-TRI6-F | AAATGCCCATTCCCTAGTTG | qRT-PCR primers of *TRI6* |
| 64 | RT-TRI6-R | ATCTCGCATGTTATCCACCCT |
| 65 | 65-CapA-F | TCACCATCACCATCACTCGAGATGTCCGATATCGAAAC | Construction of phz-65-CapA |
| 66 | 65-CapA-R | GCTCACCATCGTGGCGATGGAGCGTCGCCTCGAGCCTCCGTTGCC |
| 67 | 68-CapA-F | TCACCATCACCATCACTCGAGATGTCCGATATCGAAAC | Construction of phz-68-CapA |
| 68 | 68-CapA-R | GTTCGGGATCTTGCAGGCCGGGCGTCGCCTCGAGCCTCCGTTGCC |
| 69 | 65-Tri1-F | CGACTCACTATAGGGCGAATTGGGTACTCAAATTGTTGTGAGTAGGCCTCATAGCGAC | Construction of phz-65-Tri1 |
| 70 | 65-Tri1-R | GCTCACCATCGTGGCGATGGAGCGGTCATCCTGTACCAATTCCAATCG |
| 71 | 68-Tri1-F | CGACTCACTATAGGGCGAATTGGGTACTCAAATTGTTGTGAGTAGGCCTCATAGCGAC | Construction of phz-68-Tri1 |
| 72 | 68- Tri1-R | GTTCGGGATCTTGCAGGCCGGGCGGTCATCCTGTACCAATTCCAATCG |
